# Mechanism of COVID-19-Related Proteins in Spinal Tuberculosis: Immune Dysregulation

**DOI:** 10.3389/fimmu.2022.882651

**Published:** 2022-06-02

**Authors:** Liyi Chen, Chong Liu, Tuo Liang, Zhen Ye, Shengsheng Huang, Jiarui Chen, Xuhua Sun, Ming Yi, Chenxing Zhou, Jie Jiang, Tianyou Chen, Hao Li, Wuhua Chen, Hao Guo, Wenkang Chen, Yuanlin Yao, Shian Liao, Chaojie Yu, Shaofeng Wu, Binguang Fan, Zhaoping Gan, Xinli Zhan

**Affiliations:** ^1^ Spine and Osteopathy Ward, Guangxi Medical University First Affiliated Hospital, Nanning, China; ^2^ Department of Hematology, Guangxi Medical University First Affiliated Hospital, Nanning, China

**Keywords:** spinal tuberculosis, COVID-19, nomogram, immune cell, KEGG pathway

## Abstract

**Purpose:**

The purpose of this article was to investigate the mechanism of immune dysregulation of COVID-19-related proteins in spinal tuberculosis (STB).

**Methods:**

Clinical data were collected to construct a nomogram model. C-index, calibration curve, ROC curve, and DCA curve were used to assess the predictive ability and accuracy of the model. Additionally, 10 intervertebral disc samples were collected for protein identification. Bioinformatics was used to analyze differentially expressed proteins (DEPs), including immune cells analysis, Gene Ontology (GO) and KEGG pathway enrichment analysis, and protein-protein interaction networks (PPI).

**Results:**

The nomogram predicted risk of STB ranging from 0.01 to 0.994. The C-index and AUC in the training set were 0.872 and 0.862, respectively. The results in the external validation set were consistent with the training set. Immune cells scores indicated that B cells naive in STB tissues were significantly lower than non-TB spinal tissues. Hub proteins were calculated by Degree, Closeness, and MCC methods. The main KEGG pathway included Coronavirus disease-COVID-19. There were 9 key proteins in the intersection of COVID-19-related proteins and hub proteins. There was a negative correlation between B cells naive and RPL19. COVID-19-related proteins were associated with immune genes.

**Conclusion:**

Lymphocytes were predictive factors for the diagnosis of STB. Immune cells showed low expression in STB. Nine COVID-19-related proteins were involved in STB mechanisms. These nine key proteins may suppress the immune mechanism of STB by regulating the expression of immune genes.

## 1 Introduction

TB spreads all over the world, with high morbidity and mortality, causing a huge economic burden to society (1). STB is the most common form of extrapulmonary TB. Typical clinical symptoms of STB include back pain, fever, night sweats, fatigue, weight loss and other symptoms of TB poisoning (2, 3). In addition, STB is often accompanied by complications, including spinal instability, abscesses, spinal deformity and neurologic deficits (4). TB lesions destroy the vertebral bodies, intervertebral discs, and affect the height of the intervertebral space. In severe cases, the lesions compress the spinal cord and nerves, and surgery is required to relieve the problem. However, after decades even hundreds of years of efforts by scientists, the mechanism of Mycobacterium TB (M. TB)-induced destruction of intervertebral discs in STB has not been clarified.

Macrophages provided a site for M. TB to replicate and exhibited pro- and anti-inflammatory responses during early bacterial clearance (5, 6). Bacteria-induced release of tumor necrosis factor (TNF) caused programmed death of pathogenic macrophages through a mitochondrial-lysosomal-endoplasmic reticulum circuit (7). The function of M. TB in macrophages was inseparable from the regulation of immune cells, which were divided into T lymphocytes and B lymphocytes. B cells first appeared abnormal in active pulmonary TB but returned to normal after the control of M. TB (8, 9). The rapid accumulation of innate lymphoid cells in the lungs following M. TB infection was consistent with the accumulation of alveolar macrophages (10). Although the function of immune cells in M. TB infection of pulmonary TB was more reported, the role of immune cells in TB intervertebral disc tissue was less reported.

Since the outbreak of coronavirus disease 2019 (COVID-19) in 2019, it had rapidly spread to countries around the world, causing huge economic burdens to countries and societies (11). With the advancement of scientific research, severe acute respiratory syndrome coronavirus 2 (SARS-CoV-2) was found to be the culprit of the COVID-19 pandemic (12). Further research found that PGLYRP4 and HEPHL1 were also identified, which were novel transcriptional signature genes associated with SARS-CoV-2 virus (13). Pulmonary and extrapulmonary TB had been reported in COVID-19 patients with TB, with the former accounting for 73% (14). Isolated extrapulmonary TB in COVID-19 patients had been reported to occur in various locations, including bone, spinal in location, lymph node, genetourinary, larynx, gastrointestinal, CNS, and peritoneal (15, 16). The study by Rebecca et al. showed 333 fewer TB cases were notified between April 2020 and December 2020, compared to what would have been expected in the absence of the COVID-19 epidemic (17). Currently, 80 patients co-infected with TB and COVID-19 had been reported from 9 different countries (14). The results of Tadolini et al. showed that TB patients with COVID 19 had similar predisposing factors to those without COVID 19, including alcohol abuse, COPD, diabetes, liver disease, smoking, HIV and renal failure (15). However, is TB itself a predisposing factor for COVID 19? Or is the gene or protein of TB associated with COVID 19? Further research is required.

In this article, we analyzed the predictive role of immune cells in STB by collecting clinical data. In addition, we performed bioinformatics analysis of DEPs to explore the association of key proteins in the Coronavirus disease-COVID-19 KEGG pathway and immune mechanism in STB.

## 2 Materials and Methods

### 2.1 Patients and Data Collection

**Figure 1:**
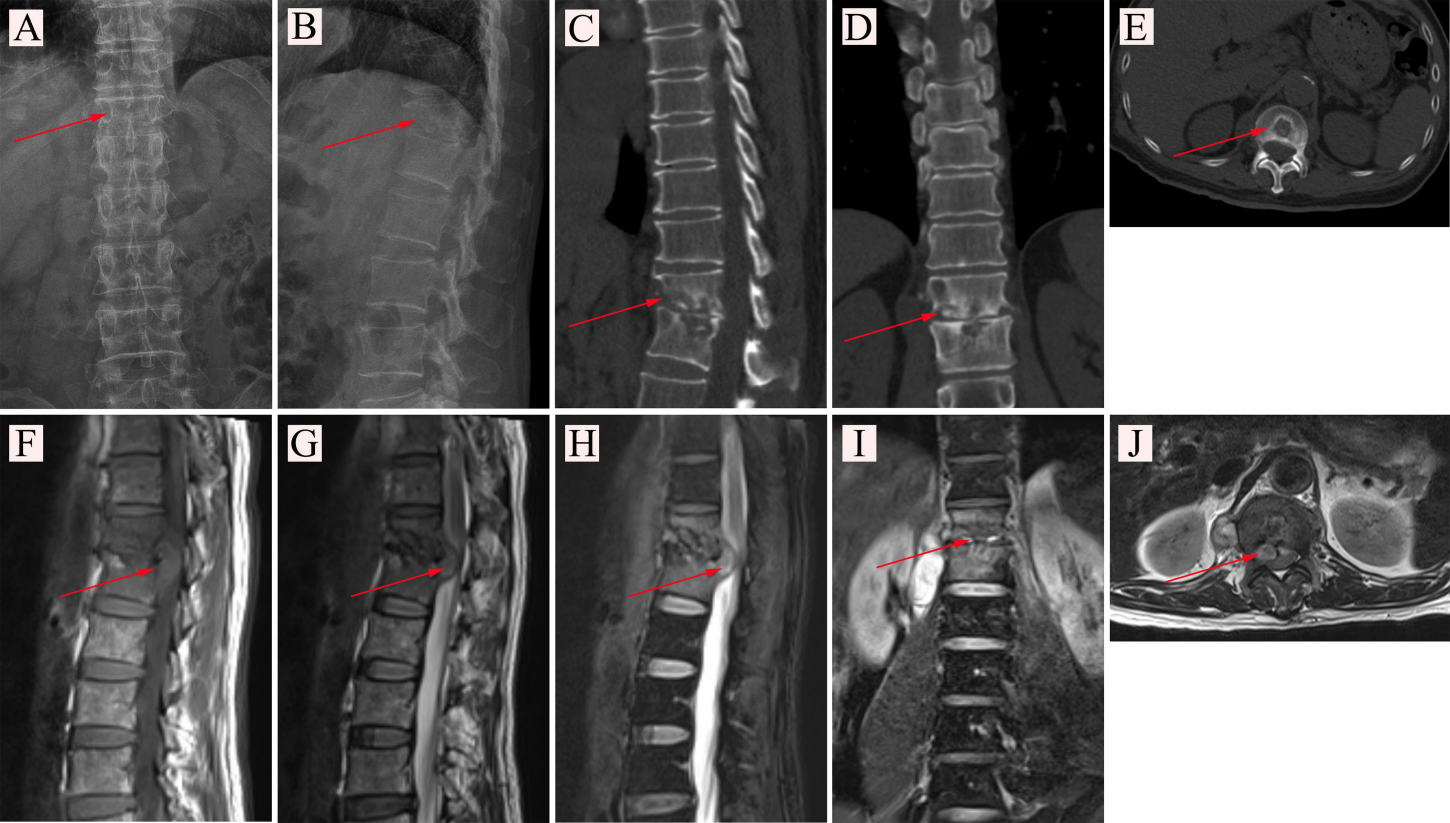
**(A, B)** X-ray examination of a patient with spinal tuberculosis. The arrow points to the location of the lesion. **(A)** X-ray image in posterior-anterior position. **(B)** X-ray image in lateral position. **(C–E)** CT examination of a patient with spinal tuberculosis. The arrow points to the location of the lesion. **(C)** CT image in sagittal position. **(D)** CT image in coronal position. **(E)** CT image in cross-section. **(F–J)** MRI examination of a patient with spinal tuberculosis. The arrow points to the location of the abscess. **(F)** MRI image in the sagittal T1 sequence. **(G)** MRI image in the sagittal T2 sequence. **(H)** MRI image of T2 lipid compression sequence in sagittal position. **(I)** MRI image in coronal position. **(J)** MRI image in cross-section.

**Figure 2:**
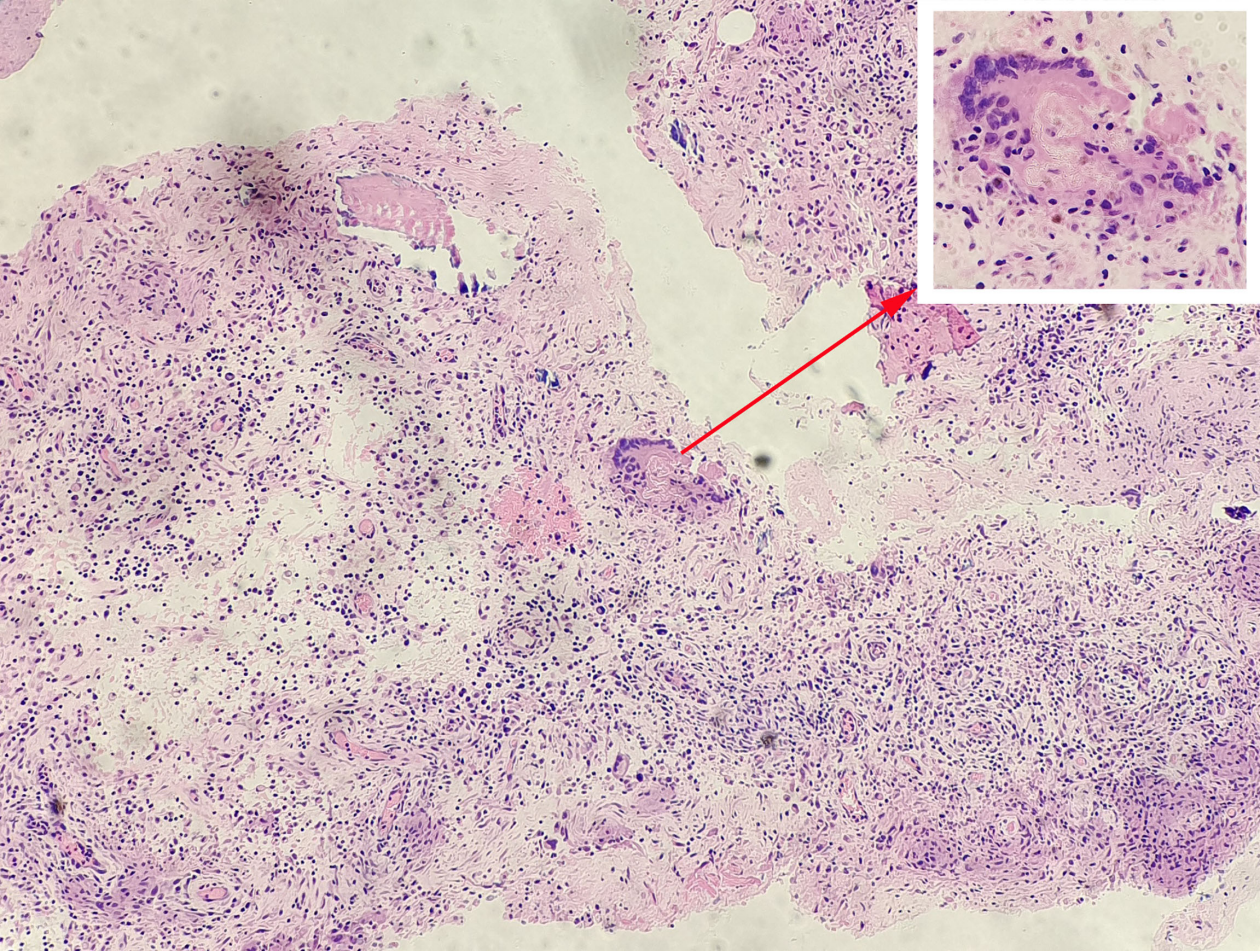
Pathological examination of a patient with spinal tuberculosis. Tuberculosis granulomas were observed in tissue sections under a microscope and consisted mainly of dermoid cells, multinucleated giant cells, and lymphocytes. The arrow points to the tuberculosis granulomas and the Langerhans multinucleated giant cells.

Clinical data of 600 patients in the training set were collected from June 2012 to March 2021. Additionally, clinical data of 400 patients were collected as an external validation set from June 2012 to December 2021. A total of 1000 clinical cases were collected from our hospital electronic case database, including 496 STB cases (**Figure 1**) and 504 non-TB spinal cases. The diagnosis of STB relied on pathological examination (**Figure 2**). General patient information was collected, including gender, age, and body mass index (BMI). Patient blood test results were collected, including erythrocyte sedimentation rate (ESR), C-reactive protein (CRP), white blood cells, hemoglobin, platelets, neutrophils, the percentage of neutrophils, lymphocytes, the percentage of lymphocytes, monocytes, and the percentage of monocytes. This project obtained the written consent of all patients and was approved by the hospital ethics committee.

### 2.2 Samples Collection

Ten intervertebral disc nucleus pulposus tissues were collected, including 5 cases of STB nucleus pulposus and 5 cases of non-STB nucleus pulposus. All tissues were cleaned up with phosphate-buffered solution (PBS, Solarbio, Beijing, China) immediately after removal during surgery. The tissues were then placed in liquid nitrogen and transferred to a -80°C freezer for storage. Clinical data of 10 patients were also collected, including the Japanese Orthopaedic Association Scores (JOA) score, Oswestry Disability Index (ODI) score, and VAS score.

### 2.3 Protein Experimental Procedures

#### 2.3.1 Protein Extraction

The tissue working solution was mixed and placed on ice to cool. The working solution was PMSF (Phenylmethylsulfonyl fluoride) (Sigma-Aldrich, St.Louis, MO, USA), RIPA lysis buffer (modified, Kangchen Biotech, Shanghai, China), mixed with Protease inhibitor cocktail (Kangchen Biotech, Shanghai, China). Take 1000ul working solution into a 1.5mL EP centrifuge tube, and then add 100mg nucleus pulposus tissue. The nucleus pulposus tissue was fully lysed using ice bath sonication. The EP tube was centrifuged for 15 minutes in a high-speed centrifuge with a speed of 14,000 g at 4°C. The centrifuged supernatant was transferred to a new EP tube.

#### 2.3.2 Protein Concentration

Standard protein solutions of 5 gradient concentrations were prepared. The BCA working solution was prepared according to the BCA protein reagent instructions. The BCA working solution was mixed with the standard protein solution and incubated at 37°C for 30 minutes. The microplate reader detects the absorbance at 562nm wavelength. The protein concentration of the samples was calculated by plotting a standard curve.

#### 2.3.3 Acetone Precipitation

100 μg of supernatant protein solution was taken from 10 samples and diluted to 1 mg/ml with RIPA lysis buffer. 4-6 volumes of acetone were added to the EP tube. The EP tubes were shaken at -20°C for 30 minutes and then centrifuged at 10,000g at 4°C. After centrifugation, the supernatant was removed, and 200 μL of acetone was added to the pellet and washed twice.

#### 2.3.4 Trypsinized Protein

200ul of 100mM ABC (ammonium bicarbonate) (Sigma-Aldrich, St. Louis, MO, USA) with 1% SDC (sodium deoxycholate, Sigma-Aldrich, St. Louis, MO, USA) was added to the protein pellet. The protein precipitate was fully dissolved using ice bath sonication. 5mM disulfide bond reducing agent TCEP (tris 2-carboxyethyl phosphine) (Sigma-Aldrich, St. Louis, MO, USA) was added to each solubilized sample. 10 mmol of indoleacetic acid (Sigma-Aldrich, St. Louis, MO, USA) was added to the EP tube. 2ug trypsin solution (protein:trypsin=50:1) was added to the EP tube containing each sample. The solution in the EP tube was mixed and incubated at 37°C for 8 hours.

#### 2.3.5 Extract Polypeptide Liquid

2% TFA (Trifluoroacetic Acid, HPLC) (Sigma-Aldrich, St. Louis, MO, USA) was added to the mixed samples to precipitate SDC in the samples. After high-speed centrifugation, the supernatant was placed in a new EP tube, and TFA was added again to precipitate the peptides. After repeating the same operation 2 times, the supernatants extracted from the 3 operations were mixed. The mixed supernatant was centrifuged again at high speed for 20 minutes. The supernatant after centrifugation was the sample polypeptide.

#### 2.3.6 Desalting of Peptides

0.1% FA (formic acid) (LC-MS, Sigma-Aldrich, St.Louis, MO, USA), 2% ACN (Acetonitrile) (LC-MS, JT Baker, PA, USA), and H_2_O were prepared as buffers A, while buffer B contains only the first two components. The C18 (3M) (Sigma-Aldrich, St. Louis, MO, USA) analytical column was rinsed with buffer A/B, respectively. The steps were repeated once. The eluate was collected after low-speed centrifugation. The eluate was lyophilized under vacuum. Buffer A was added to the lyophilized eluate to reconstitute the peptide to 1 ug/ul for LC-MS/MS detection.

#### 2.3.7 Liquid Chromatography-Tandem Mass Spectrometry Detection (LC-MS/MS)

A 2 μg peptide sample was taken from each component for separation by an ultra-high performance liquid chromatography system EASY-nLC1200 (Thermo Fisher Scientific, USA). Detection was performed using an online mass spectrometer Q-Exactive (Thermo Fisher Scientific, USA). Three technical replicates were performed for each protein sample.

#### 2.3.8 MaxQuant Analysis and LFQ Quantification

MaxQuant (Version 1.5.6.0) was used to analyze raw LC-MS/MS detection data to obtain peptide sequence information. The sequence information was entered into the UniProt protein database (uniport human 20181016 iso-ZL) for forward and reverse sequence searches and protein identification. The quantitation type was set to label-free quantification (LFQ) with inter-run detection.

### 2.4 Clinical Data Analysis

LASSO regression was used to analyze the training set data to obtain non-zero coefficient parameters. These parameters were included in logistic regression analysis, and then parameters with P<0.05 were selected to construct nomograms. C-index, calibration curve, ROC curve, and DCA curve were used to evaluate the nomogram. The same parameters were chosen to construct the nomogram from the validation set data. The C-index, calibration curve, ROC curve and DCA curve were also used to evaluate the validation set nomogram.

### 2.5 Immune Cells Score

We calculated 22 immune cells scores for each sample based on the R language “CIBERSORT” package.

### 2.6 Bioinformatics Analysis

#### 2.6.1 Calculate DEPs

We log2-transformed raw protein expression data and calculated DEPs using the “limma” package. Proteins with |Log Fold change (FC) |>2 and P.Value <0.05 were identified as DEPs.

#### 2.6.2 GO Enrichment Analysis

The “enrichGO” package was run in the R language for functional enrichment analysis, including molecular function (MF), biological process (BP) and cellular component (CC) analysis. We also use the R language to run the “enrichKEGG” package for KEGG analysis. The 5 most protein counts enrichment analysis results were selected for plotting.

#### 2.6.3 PPI Network Construction

PPI Network analysis was performed using the online protein database(https://string-db.org/). All DEPs were included in the calculation. The online database sets composite score >0.4. The exported results were imported into Cytoscape software for further analysis.

#### 2.6.4 Key Proteins

We identified key proteins with reference to previous research methods (13). Cytoscape software was used to calculate the top 30 hub proteins by Degree, Closeness, and MCC methods from PPI networks, respectively. Key proteins were screened from the co-intersection of hub proteins and the proteins of the Coronavirus disease-COVID-19 pathway.

### 2.7 Statistical Analysis

This paper used R software (version 4.1) for statistical analysis. Cytoscape software (version 3.9) was used to calculate hub proteins. Measurement data were expressed as mean ± SD. P <0.05 was considered statistically significant.

## 3 Results

### 3.1 Lymphocytes as Predictors of STB

#### 3.1.1 Patient Characteristics

A total of 1000 patients’ clinical data were collected in this paper, including 600 cases for constructing the training set and 400 cases for external validation. The clinical characteristics of the STB group and the non-TB spinal group were shown in **Table 1**.

**Table 1:** Clinical characteristics of patients in the spinal tuberculosis and the non-spinal tuberculosis.

Characteristics	Non-tuberculosis spinal disease (N = 504)	Spinal tuberculosis (N = 496)	P-value
Gender			
female	73 (14 %)	199 (40 %)	<0.001
male	431 (86 %)	297 (60 %)	
Age			
Mean ± SD	35.0 ± 10.5	50.2 ± 17.5	<0.001
BMI			
<18.5	29 (6 %)	130 (26 %)	<0.001
18.5~23.9	379 (75 %)	317 (64 %)	
>23.9	96 (19 %)	49 (10 %)	
ESR			
Mean ± SD	30.1 ± 22.0	41.7 ± 30.6	<0.001
CRP			
Mean ± SD	29.4 ± 38.2	30.1 ± 39.5	0.766
White blood cells			
Mean ± SD	8.25 ± 2.09	7.31 ± 2.82	<0.001
Hemoglobin			
Mean ± SD	132 ± 18.0	120 ± 17.5	<0.001
Platelets			
Mean ± SD	317 ± 98.1	305 ± 103	0.043
Percentage of neutrophils			
Mean ± SD	0.627 ± 0.0986	0.632 ± 0.122	0.466
Neutrophils			
Mean ± SD	5.26 ± 1.87	4.76 ± 2.54	<0.001
Percentage of lymphocytes			
Mean ± SD	0.268 ± 0.0902	0.235 ± 0.105	<0.001
Lymphocytes			
Mean ± SD	2.13 ± 0.740	1.61 ± 0.827	<0.001
Monocytes			
Mean ± SD	0.636 ± 0.238	0.651 ± 0.280	0.374
Percentage of monocytes			
Mean ± SD	0.0782 ± 0.0250	0.0974 ± 0.0600	<0.001

#### 3.1.2 Building a Nomogram Predictive Model

**Figure 3:**
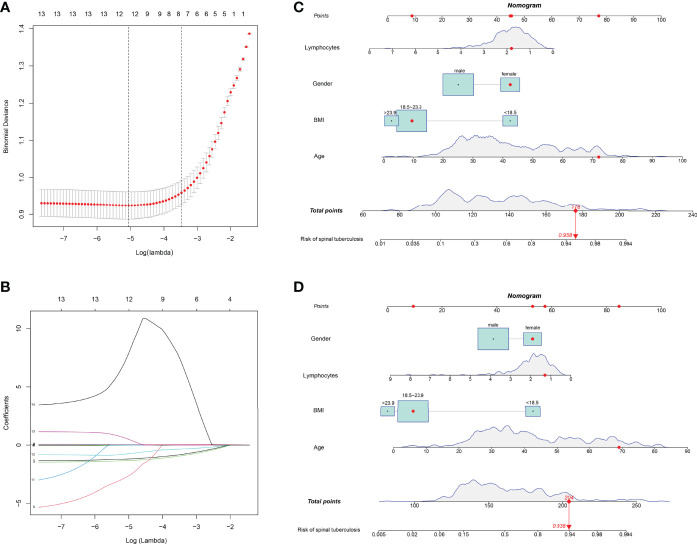
Parameters were selected to build LASSO regression and nomogram models. **(A)** The binomial deviation was plotted through the optimal log (Lambda) value. **(B)** 12 non-zero coefficient parameters are screened by the optimal log (Lambda) value. **(C)** The nomogram predicting the risk of spinal tuberculosis was plotted in the training set. **(D)** The nomogram predicting the risk of spinal tuberculosis was plotted in the external validation set.

All parameters were included in LASSO regression analysis. The binomial deviation was plotted through the optimal log (Lambda) value (**Figure 3A**). Coefficient plots for LASSO regression were also plotted by log (Lambda) values. 12 non-zero coefficient parameters are screened by the optimal log (Lambda) value (**Figure 3B**). These parameters with non-zero coefficients were included in logistic regression analysis. The results showed statistical differences in 4 parameters, including gender, age, BMI, and lymphocytes (**Table 2**). These four parameters were then chosen to construct the nomogram. The nomogram of the training set predicted the risk of STB ranging from 0.1 to 0.994 (**Figure 3C**). The nomogram of the external validation set also predicted risk in the same range from 0.1 to 0.994 (**Figure 3D**).

**Table 2:** Multivariate logistic regression analysis.

Characteristics	OR	95%CI	P-value
Gender(male)	0.260	0.147-0.451	<0.001
Age	1.076	1.058-1.096	<0.001
BMI			
18.5~23.9	0.081	0.038-0.163	<0.001
>23.9	0.049	0.019-0.117	<0.001
Lymphocytes	0.332	0.179-0.599	<0.001

Female in gender and BMI <18.5 were used as reference values for analysis.

#### 3.1.3 Evaluating the Nomogram Prediction Model

**Figure 4:**
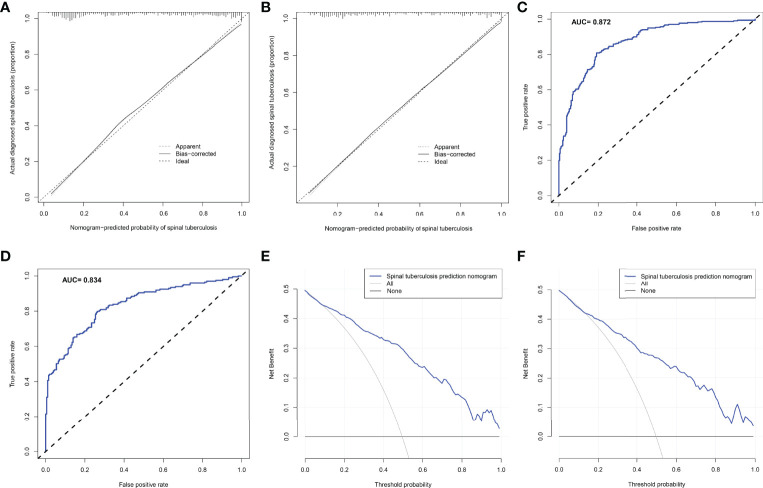
The predictive ability and accuracy of the nomogram model was evaluated. **(A)** The calibration curve for the training set showed that the nomogram predicted risk of spinal tuberculosis was highly consistent with the actual diagnosis of spinal tuberculosis. **(B)** The calibration curve for the external validation set showed that the predicted and actual values were also highly consistent. **(C)** The ROC curve of the training set showed that the AUC of the nomogram model for predicting spinal tuberculosis was 0.862. **(D)** The ROC curve of the external validation set calculated the AUC to be 0.834. **(E)** The DCA curve of the training set showed that the net benefit of the predictive model ranged from 0.01 to 0.99. **(F)**, The DCA curve of the external validation set showed that the net benefit of the predictive model also ranged from 0.01 to 0.99.

The C-index of the nomogram constructed from the training set was 0.872 (95% CI: 0.845-0.899). The C-index of the training set calculated by the internal validation method was 0.866. The nomogram C-index of the external validation set was also as high as 0.848 (95%CI: 0.811-0.885). The calibration curve for the training set showed that the nomogram predicted risk of STB was highly consistent with the actual diagnosis of STB (**Figure 4A**). Interestingly, the calibration curve for the external validation set showed that the predicted and actual values were also highly consistent (**Figure 4B**). The ROC curve of the training set showed that the AUC of the nomogram model for predicting STB was 0.862 (**Figure 4C**). Similarly, the ROC curve of the external validation set calculated the AUC to be 0.834 (**Figure 4D**). The DCA curve of the training set showed that the net benefit of the predictive model ranged from 0.01 to 0.99(**Figure 4E**), which was consistent with that of the external validation set (**Figure 4F**).

#### 3.1.4 Lymphocytes Expression

**Figure 5:**
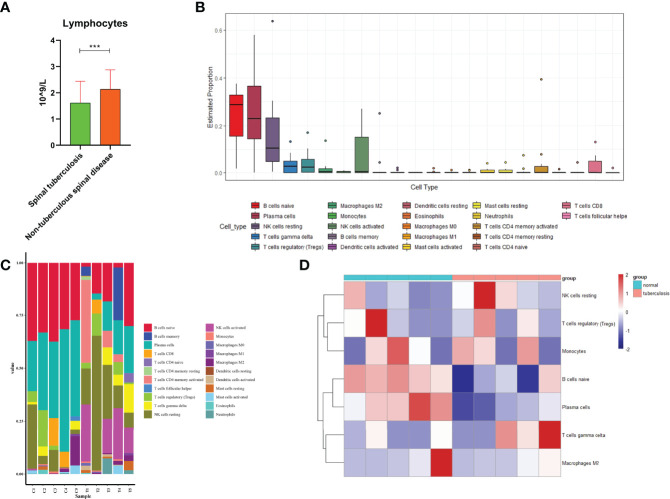
Immune cells analysis. **(A)** Comparison of lymphocytes in spinal tuberculosis and nontuberculous spinal disease. **(B)** The estimated distribution of 22 immune cells was plotted. **(C)** The expression ratio of 22 immune cells in each sample was plotted. **(D)** Heatmap showed expression of immune cells in spinal tuberculosis and non-spinal tuberculosis tissues and showed that the expression of B cells naive former tissues was lower than that in the latter tissues. ***:P<0.001.

We found that the lymphocytes were 1.61 ± 0.83 X10^9^/L in STB and 2.13 ± 0.74 X 10^9^/L in non-TB spinal disease(**Figure 5A**). There was a statistically significant difference between the two groups(P<0.001).

### 3.2 Immune Cell Analysis

A total of 1965 proteins were identified in 10 intervertebral disc samples, including 5 STB disc samples and 5 non-TB spinal disc samples. The estimated distribution of 22 immune cells showed that the three cells with the highest distribution in the sample were B cells naive, platelet cells, and NK cells resting (**Figure 5B**). Immune cells for each sample were displayed by histograms and B cells naive had higher expression levels in each sample (**Figure 5C**). Heatmap of 10 clinical samples showed a clear degree of clustering of B cells naive in spinal tissue and non-TB spinal tissue (**Figure 5D**).

**Figure 6:**
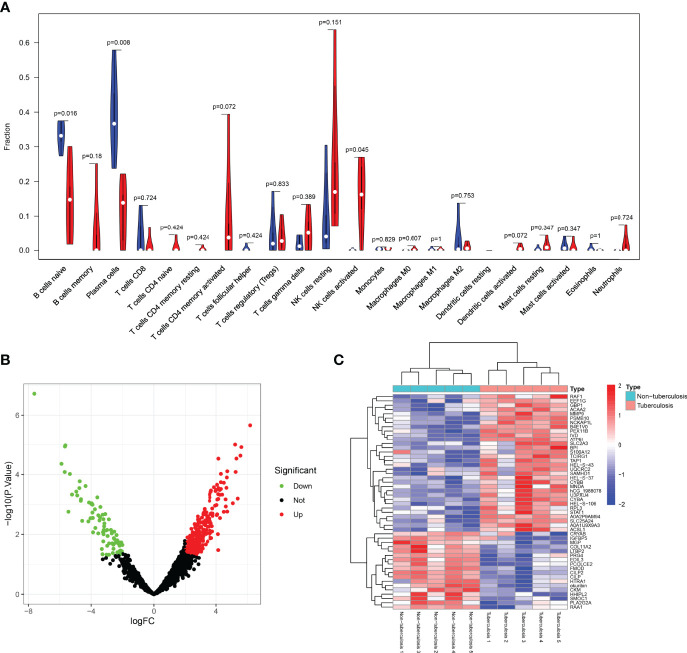
Violin, volcano, and heatmap were plotted. **(A)** The comparison of 22 immune cell scores in spinal tuberculosis and non-spinal tuberculosis was plotted by the violin plot. There were statistical differences among the three types of immune cells, including B cells naive, platelet cells, and NK cells activated. **(B)** The volcano plot showed that the differential proteins were divided into up-regulated and down-regulated proteins. **(C)** The heatmap clearly showed the clustering of the top 50 proteins in spinal tuberculosis tissues and non-spinal tuberculosis tissues.

The comparison of 22 immune cells scores in the STB group and the non-TB spinal group was shown in the violin plot (**Figure 6A**). There were statistical differences among the three types of immune cells, including B cells naive(P=0.016), platelet cells (P=0.008), and NK cells activated (P=0.045).

### 3.3 Protein Bioinformatics Analysis

#### 3.3.1 DEPs Analysis

The sample proteins were analyzed by LC-MS/MS, and 1965 quantifiable proteins were identified. The conditions for calculating differential proteins were set as logFoldChange>2 and P.Value<0.05. A total of 350 DEPs were screened, including 86 down-regulated proteins and 264 up-regulated proteins. The volcano plot showed that the DEPs were divided into up-regulated and down-regulated proteins (**Figure 6B**). The heatmap clearly showed the clustering of the top 50 proteins in STB tissue and non-TB spinal tissue (**Figure 6C**).

#### 3.3.2 GO Functional Enrichment Analysis

**Figure 7:**
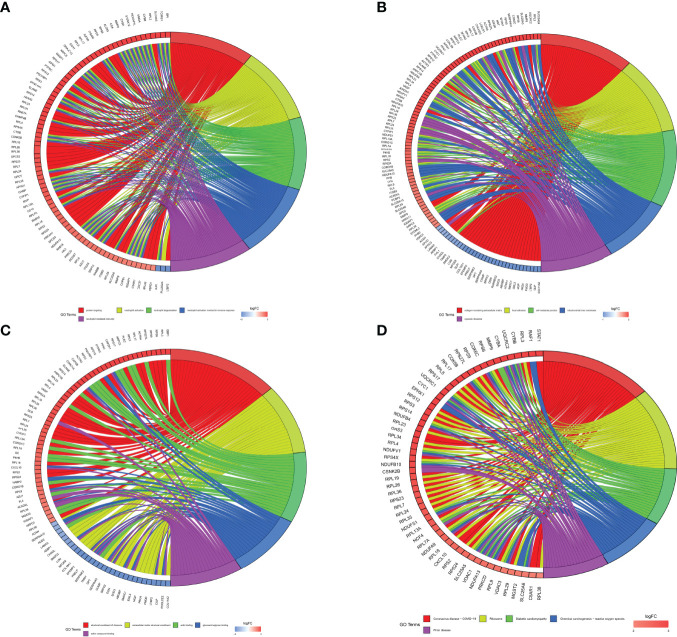
GO and KEGG pathway enrichment analyses were plotted. **(A)** BP functional enrichment had 284 entries. The 5 entries with the most enriched protein counts were plotted. **(B)** CC functional enrichment had 284 entries. The 5 entries with the most enriched protein counts were plotted. **(C)** MF functional enrichment had 284 entries. The 5 entries with the most enriched protein counts were plotted. **(D)** KEGG pathway enrichment had 284 entries. The 5 entries with the most enriched protein counts were plotted.

All DEPs were selected to GO functional enrichment analysis, and the screening conditions were set as p.value and p.adjust were both less than 0.05. BP functional enrichment had 284 entries. The 5 entries with the most enriched protein counts were plotted, including protein targeting, neutrophil activation, neutrophil degranulation, neutrophil activation involved in immune response, and neutrophil mediated immunity (**Figure 7A**). The CC functional enrichment had 106 entries. The 5 entries with the most enriched protein counts were plotted, including collagen-containing extracellular matrix, focal adhesion, cell-substrate junction, mitochondrial inner membrane and cytosolic ribosome (**Figure 7B**). The MF functional enrichment had 55 entries. The 5 entries with the most enriched protein counts were plotted, including structural constituent of ribosome, extracellular matrix structural constituent, actin binding, glycosaminoglycan binding and sulfur compound binding (**Figure 7C**).

#### 3.3.3 KEGG Pathway Enrichment Analysis

All DEPs were selected for KEGG pathway enrichment analysis, and the screening conditions were set as p.value and p.adjust were both less than 0.05. 26 KEGG pathways were enriched. The 5 KEGG pathways with the most protein counts were plotted, including Coronavirus disease - COVID-19, ribosome, diabetic cardiomyopathy, chemical carcinogenesis - reactive oxygen species and prion disease (**Figure 7D**). Coronavirus disease - COVID-19 was enriched for the most proteins by the KEGG pathway, including 34 proteins that might play important roles in STB.

#### 3.3.4 PPI Network Construction

**Figure 8:**
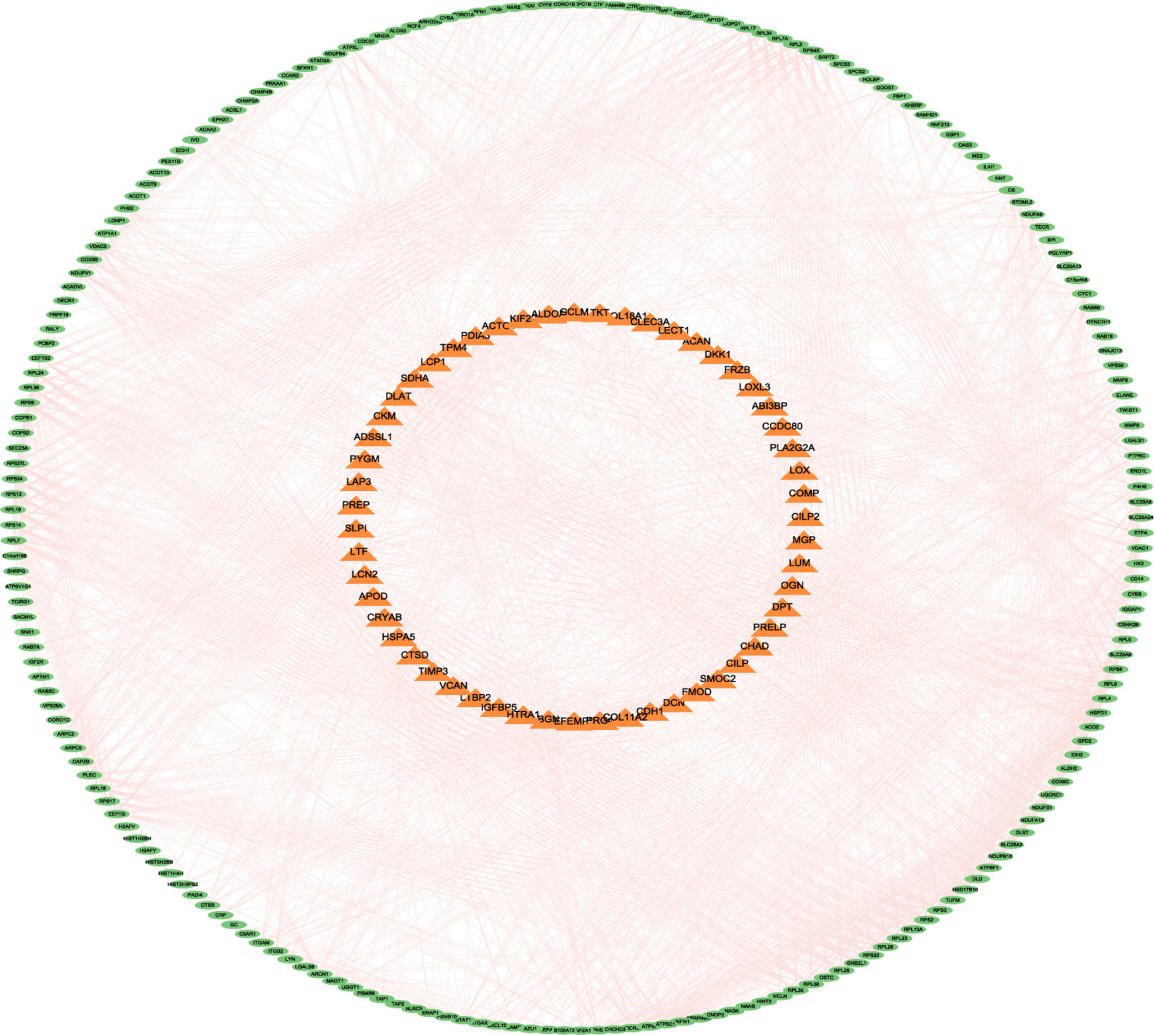
The differential proteins were divided into up-regulated proteins and down-regulated proteins by constructing a PPI network map. Outer circle proteins indicated up-regulated proteins and inner circle proteins indicated down-regulated proteins.

All DEPs were included in the construction of the PPI network analysis. Furthermore, Cytoscape software was used for drawing and analysis. The differential proteins were divided into up-regulated proteins and down-regulated proteins by constructing a PPI network map. Outer circle proteins indicated up-regulated proteins and inner circle proteins indicated down-regulated proteins (**Figure 8**).

#### 3.3.5 Identification of Key Proteins

**Figure 9:**
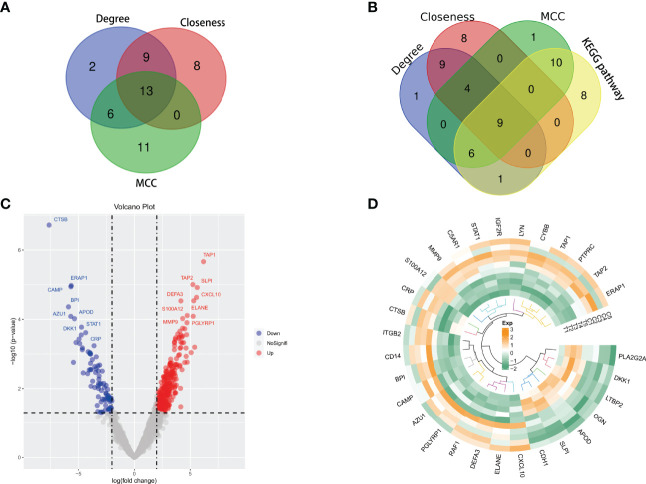
**(A, B)** Venn diagrams were plotted. **(A)** Cytoscape software calculated the hub 30 key proteins by Closeness, Degree, and MCC methods, respectively. A total of 13 hub proteins were obtained by the intersection of three methods. **(B)** There were 9 key proteins in the intersection of the hub proteins calculated by Cytoscape and the proteins of the Coronavirus disease-COVID-19 pathway. **(C, D)** Differential analysis of immune-related genes. **(C)** 30 immune-related genes include 7 down-regulated genes and 23 up-regulated genes. **(D)** The circus heatmap clearly showed the clustering of all the immune-related genes in STB tissue and non-TB spinal tissue.

Cytoscape software calculated the hub 30 key proteins by Closeness, Degree, and MCC methods, respectively. A total of 13 hub proteins were obtained by the intersection of three methods (**Figure 9A**). These proteins again underwent a secondary Venn diagram intersection with proteins of the KEGG pathway Coronavirus disease - COVID-19. There were 9 key proteins in the intersection of the hub proteins calculated by Cytoscape and the COVID-19-related proteins (**Figure 9B**). These 9 key proteins were RPS6, RPL23, RPS9, RPL5, RPL13A, RPS3, RPL4, RPL9 and RPL19.

### 3.4 Immune-Related Genes

Among all DEPs, we found 30 immune-related genes, including 7 down-regulated genes and 23 up-regulated genes (**Figure 9C**). The circus heatmap clearly showed the clustering of all the immune-related genes in STB tissue and non-TB spinal tissue (**Figure 9D**).

### 3.5 Correlation Between COVID-19-Related Proteins and Immune Regulation

**Figure 10:**
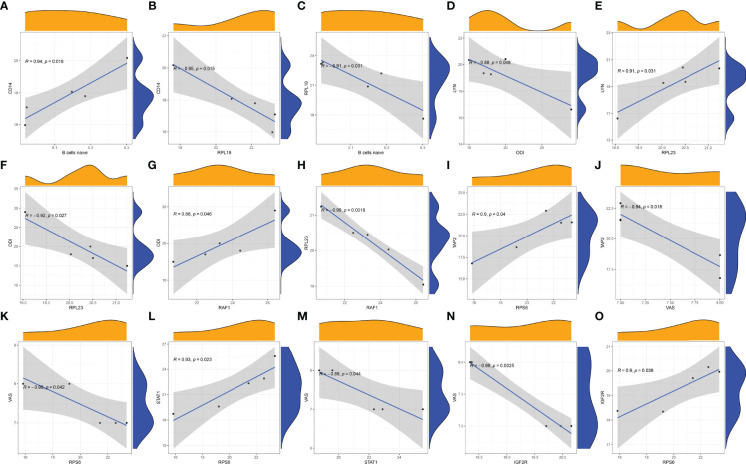
Correlation analysis was plotted. **(A)** Correlation analysis of B cells naive and CD14. **(B)** Correlation analysis of RPL19 and CD14. **(C)** Correlation analysis of B cells naive and RPL19. **(D)** Correlation analysis of LYN and ODI. **(E)** Correlation analysis of RPL23 and LYN. **(F)** Correlation analysis of RPL23 and ODI. **(G)** Correlation analysis of RAF1 and ODI. **(H)** Correlation analysis of RPL23 and RAF1. **(I)** Correlation analysis of TAP2 and RPS6. **(J)** Correlation analysis of TAP2 and VAS. **(K)** Correlation analysis of RPS6 and VAS. **(L)** Correlation analysis of RPS6 and STAT1. **(M)** Correlation analysis of STAT1and VAS. **(N)** Correlation analysis of IGF2R and VAS. **(O)** Correlation analysis of RPS6 and IGF2R.

In STB tissue, we found that the immune gene CD14 was positively correlated with B cells naive (**Figure 10A**). However, the COVID-19-related protein RPL19 was negatively correlated with CD14 (**Figure 10B**) and B cells naive (**Figure 10C**). RPL19 may inhibit the expression of immune B cells naive in STB by regulating the expression of CD14. The immune gene LYN was negatively correlated with ODI (**Figure 10D**). There was a positive correlation between COVID-19-related protein RPL23 and LYN (**Figure 10E**). However, RPL23 was negatively correlated with ODI (**Figure 10F**). There was a positive correlation between the immune gene RAF1 and ODI (**Figure 10G**). However, RPL23 was negatively correlated with RAF1(**Figure 10H**).RPL23 may reduce ODI score in STB by regulating the expression of LYN and RAF1. There was a positive correlation between COVID-19-related protein RPS6 and TAP2(**Figure 10I**). The immune gene TAP2 was negatively correlated with VAS (**Figure 10J**). Similarly, RPS6 was negatively correlated with VAS (**Figure 10K**). There was a positive correlation between RPS6 and the immune gene STAT1 (**Figure 10L**). However, STAT1 was negatively correlated with VAS (**Figure 10M**). The immune gene IGF2R was negatively correlated with VAS (**Figure 10N**). However, there was a positive correlation between RPS6 and IGF2R (**Figure 10O**). RPS6 may reduce the VAS score of STB patients by regulating the expression of TAP2, STAT1 and IGF2R.

**Figure 11:**
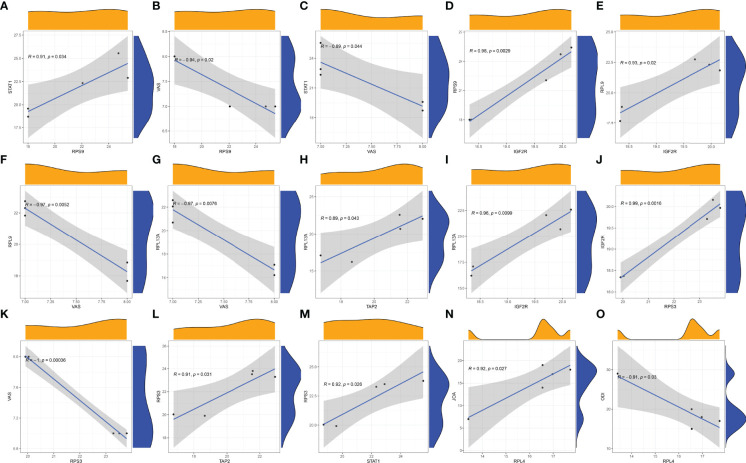
Correlation analysis was plotted. **(A)** Correlation analysis of STAT1 and RPS9. **(B)** Correlation analysis of RPS9 and VAS. **(C)** Correlation analysis of STAT1 and VAS. **(D)** Correlation analysis of IGF2R and RPS9. **(E)** Correlation analysis of RPL9 and IGF2R. **(F)** Correlation analysis of RPL9 and VAS. **(G)** Correlation analysis of RPL13A and VAS. **(H)** Correlation analysis of RPL13A and TAP2. **(I)** Correlation analysis of IGF2R and RPL13A. **(J)** Correlation analysis of IGF2R and RPS3. **(K)** Correlation analysis of RPS3 and VAS. **(L)** Correlation analysis of RPS3 and TAP2. **(M)** Correlation analysis of STAT1and RPS3. **(N)** Correlation analysis of RPL4 and JOA. **(O)** Correlation analysis of RPL4 and ODI.

There was a positive correlation between the COVID-19-related protein RPS9 and STAT1 (**Figure 11A**). There was a negative correlation between VAS and RPS9 (**Figure 11B**). Similarly, VAS was negatively correlated with STAT1 (**Figure 11C**). In addition, there was a positive correlation between RPS9 and IGF2R (**Figure 11D**). RPS9 may reduce the VAS score of STB patients by regulating the expression of STAT1 and IGF2R. There was a positive correlation between the COVID-19-related protein RPL9 and IGF2R (**Figure 11E**). However, RPL9 was negatively correlated with VAS (**Figure 11F**).RPL9 may reduce the VAS score of STB patients by regulating the expression of IGF2R. The COVID-19-related protein RPL13A was negatively correlated with VAS (**Figure 11G**). However, RPL13A positively correlated with both TAP2 (**Figure 11H**) and IGF2R (**Figure 11I**). RPL13A may reduce the VAS score of STB patients by regulating the expression of TAP2 and IGF2R. There was a positive correlation between the COVID-19-related protein RPS3 and IGF2R (**Figure 11J**). However, there was a positive correlation between RPS3 and VAS (**Figure 11K**). RPS3 was positively correlated with both TAP2 (**Figure 11L**) and STAT1 (**Figure 11M**). RPS3 may reduce the VAS score of STB patients by regulating the expression of IGF2R, TAP2 and STAT1. In addition, we found a positive correlation between the COVID-19-related protein RPL4 and JOA (**Figure 11N**). However, RPL4 was negatively correlated with ODI (**Figure 11O**). Immune genes were not involved in RPL4 regulation of JOA and ODI scores of STB patients.

**Figure 12:**
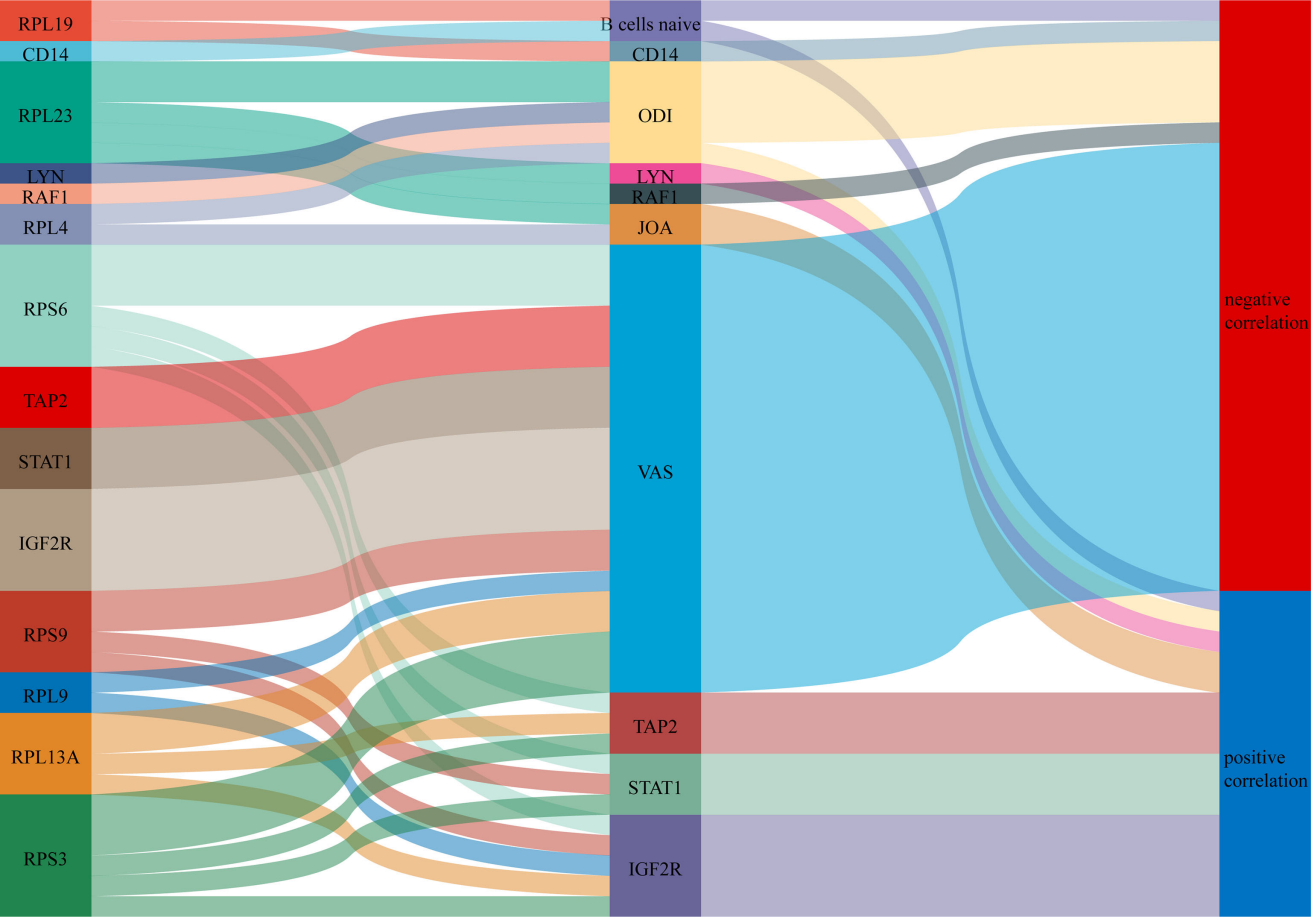
A Sankey diagram was plotted showing the relationship between COVID-19-related proteins regulating immune genes and clinical scores.

A Sankey diagram was plotted showing the relationship between COVID-19-related proteins regulating immune genes and clinical scores (**Figure 12**).

## 4 Discussion

STB was an infectious disease caused by M. TB invading the intervertebral discs and vertebral bodies. There were many methods for the diagnosis of STB, the most authoritative of which was a pathological examination. However, early STB had limitations in diagnosing without pathological examination. We collected 1000 cases of clinical data. Lymphocytes were screened by LASSO regression and Logistic regression methods. Lymphocytes were used to construct nomograms and played an important role in predicting the risk of STB. To further verify the role of immune cells in STB, we collected 10 intervertebral disc samples. A total of 1965 quantifiable proteins were identified. The results of immune cells scoring indicated that B cells naive were differentially expressed in STB. Both the B cells naive of the intervertebral disc tissue and the lymphocytes of the blood were lowly expressed in STB. DEPs were enriched to a Coronavirus disease-COVID-19 pathway by KEGG enrichment analysis. In addition, we referred to previous research strategies, which identified common key genes through enrichment analysis and modules (13). We also used the COVID-19 KEGG enrichment pathway and the three modules of MCC, Closeness, and Degree to identify key proteins.

M. TB was mainly phagocytosed by macrophages. The source of macrophages evolved from monocytes. Therefore, the expression of monocytes in the blood was usually increased in STB. However, lymphocytes act as immune cells, and M. TB suppresses the immune function of the body to avoid being killed, which might lead to suppressed immune cells expression. The study by Lv et al. found that the lymphocytes of non-TB were 1.48 ± 0.05, that of pulmonary TB was 1.31 ± 0.08, and that of extrapulmonary TB was 1.23 ± 0.06 (18). The number of lymphocytes in non-TB was significantly decreased, and the comparison was statistically significant(P=0.004). M. TB was effectively controlled after CD8+ T lymphocytes were inhibited (19). As a member of the NLR family, NLRC3 effectively inhibited the expression of CD4+ T cells and promoted M. TB infection (20). Similarly, our study found that lymphocytes in STB were significantly lower than those in the non-TB spinal group. Lymphocytes played an important role in the model predicting the risk of STB.

B lymphocytes might play a role in the immune escape of M. TB. A study by Corominas et al. showed that 35 TB patients had significantly lower B lymphocytes than 20 healthy patients (21). In M. TB -infected mice with knockout B cells, bacterial load was significantly increased compared to wild-type mice (22). However, TB granulomas developed in mice with reconstituted naive B cells, a widespread form consistent with wild-type mice (23). After M. TB activation, naive B cells secrete M. TB -specific antibodies and release cytokines, and naive B cells further develop into activated plasma cells (24). In this paper, the B cells naive of TB tissue was significantly lower than that of non-TB tissue in the immune cells score. This might be that M. TB infection suppressed the immune response of B lymphocytes, or that M. TB was activated in macrophages and stimulated the development of B cells into activated plasma cells.

In this study, the DEPs were included in the KEGG enrichment analysis, and a Coronavirus disease-COVID-19 KEGG pathway was enriched. Although there was no evidence that COVID-19 induced TB or that TB induced COVID-19, there had been reports in the literature of COVID-19 complicated with TB (25–27). The predisposing factors of COVID 19 were similar to those of TB, such as HIV, COPD, diabetes, alcohol abuse, and smoking (14, 15). In patients with COVID 19, isolated TB foci had been reported in bone, spinal in location, and other sites (15, 16). The relationship between TB and COVID 19 might be linked to BCG injection. For every 10% increase in BCG administration, there was a 10.4% reduction in COVID 19 mortality (28–30). Mortality rates from COVID 19 were lower in seven countries where BCG was mandatory, compared to countries that stopped BCG for 20 years (28, 29). However, there was no literature on how co-infection between COVID-19 and TB occurred. Whether patients with STB who carry COVID-19-related proteins are more prone to TB combined with COVID-19 under exposure to COVID-19 virus, which needs further study.

Although the TB patient data we collected did not have a confirmed diagnosis of COVID-19, a Coronavirus disease-COVID-19 pathway was identified by KEGG enrichment analysis. There was a total of 34 proteins in this pathway, including CYBB, STAT1, OAS3, C5AR1, CXCL10, and ribosomal proteins. It might be that the DEPs expressed in TB were also differentially expressed in COVID-19. The spike protein of Severe Acute Respiratory Syndrome Coronavirus 2 (SARS-CoV-2) inhibited STAT1 activation, blocked its association with the JAK1 pathway, and then inhibited the host type I interferon response (31). In addition, SARS-CoV-2 spike protein interacted with dendritic cells to activate inflammatory pathways including MAPK, AKT, STAT1, and NF-κB and promoted the release of inflammatory cytokines (32). OAS3 was identified as one of the highly conserved genes in type I interferon response, regulating IFN-I signaling, and contributing to the diagnosis of COVID-19 (33). OAS3 was also identified as predicting the severity of COVID-19 (34). CXCL10 was released along with pro-inflammatory mechanisms that promoted pulmonary fibrosis in COVID-19 patients through the CXCL10-CXCR3 signaling system and CXCL10 was identified with a poor prognosis in COVID-19 patients (35, 36). The ribosomal protein family was widely expressed in the Coronavirus disease-COVID-19 pathway. Our differential protein bioinformatics analysis identified 29 ribosomal proteins. Both mitochondrial and cytoplasmic ribosomal proteins were closely associated with SARS-CoV-2 longest non-structural protein 3 (NSP3), which played a role in RNA metabolism and immune responses (37). A large number of ribosomal proteins and viral mRNAs were involved in viral protein biosynthesis, and these proteins were important for viral replication and infection of host cells. RPL 9 and RPL 10 could be used to assess the activity of Covid-19 and even became targets for the development of Covid-19 vaccines and treatments (38).

In this paper, we found that nine key proteins related to COVID-19 were involved in the regulation of STB through immune genes. RPL19 might suppress STB immune B cells naive by regulating the immune gene CD14. The study by Wang et al. showed that RPL19 and CD14 were involved in the immune response intracellularly (39). In addition, the gene polymorphism of CD14 was a high-risk factor for STB (40). We found that RPS3 was negatively associated patient’s VAS. RPS3 synergistically enhanced DNA binding with P65 and P65-P50. Once RPS3 was knocked out, the transcriptional function of NF-κB subunit P65 was affected (41). BfrB impeded NF-κB function by inhibiting the nuclear abundance of RPS3, resulting in reduced host innate immunity against M. TB infection (42). RPS6 had involved in the transformation of M. TB S18 ribosomal proteins along with Zinc (43). In this paper, RPS6 could also regulate the expression of immune genes TAP2, STAT1 and IGF2R to suppress VAS in patients.

In addition, the systemic endocrine system may also be involved in the immune dysregulation of COVID-19 (44). Spinal tuberculosis usually suffered from vertebral body destruction with coexisting osteogenic and osteoclastic processes (45, 46). The RANK-RANKL pathway not only played an important role in regulating the mechanism of osteoclasts (47). In addition, RANK-RANKL was also involved in immune regulation to modulate lymph node maturation, immunosuppression and presenting cell activity, and further participated in lymphocyte self-tolerance (48).

However, this study still had some limitations. (1) The clinical data was a single-center study, and a multi-center study was required to validate the nomogram model. (2) Tissue samples for COVID-19 with STB could not be collected for experimental verification.

## 5 Conclusion

Lymphocytes were predictive factors for the diagnosis of STB. Immune cells showed low expression in STB. Nine COVID-19-related proteins were involved in STB mechanisms, including RPS6, RPL23, RPS9, RPL5, RPL13A, RPS3, RPL4, RPL9, and RPL19. These nine key proteins may suppress the immune mechanism of STB by regulating the expression of immune genes.

## Data Availability Statement

The data presented in the study are deposited in the NCBI repository, accession number PRJNA841580.

## Ethics Statement

The studies involving human participants were reviewed and approved by the ethics committee of The First Affiliated Hospital of Guangxi Medical University. The patients/participants provided their written informed consent to participate in the study.

## Author Contributions

LC wrote the article and prepared figures and tables. CL, this author contributed equally to this work and should be considered co-first authors. ZG and XZ have shared contributions and should be considered co-corresponding authors. All authors reviewed the article. All authors contributed to the article and approved the submitted version.

## Funding

This work was sponsored by the National Natural Science Foundation of China (81560359); National Natural Science Foundation of China (81860393). Funding bodies had no role in the study design, collection, analysis, and interpretation of the data or in writing the manuscript.

## Conflict of Interest

The authors declare that the research was conducted in the absence of any commercial or financial relationships that could be construed as a potential conflict of interest.

## Publisher’s Note

All claims expressed in this article are solely those of the authors and do not necessarily represent those of their affiliated organizations, or those of the publisher, the editors and the reviewers. Any product that may be evaluated in this article, or claim that may be made by its manufacturer, is not guaranteed or endorsed by the publisher.
